# Supplementary material for: Identifying cell-type-specific spatially variable genes with ctSVG
Source: Genome Biol. 2025 Dec 8;26:416. doi: 10.1186/s13059-025-03870-6 (PMC12683811; doi:10.1186/s13059-025-03870-6)
Supplement: Supplementary file 1 — Additional file 1: Fig. S1. Spearman correlation coefficient between the overall gene rankings obtained by the default ctSVG and those obtained by ctSVG with different levels of cell nuclei expansion. Fig. S2. Spearman correlation coefficient between the overall gene rankings obtained by ctSVG from the original data and those from the perturbed data. Fig. S3. Number of cells in each Visium HD dataset after rectangular subsetting. Fig. S4. Computational time and peak memory usage of ctSVG across seven real Visium HD datasets. Fig. S5. Cell clustering and cell type annotation in the mouse embryo tissue. Fig. S6. Expression level of marker genes in the mouse embryo tissue. Fig. S7. Cell clustering and cell type annotation in the human colon cancer tissue. Fig. S8. Expression level of marker genes in the human colon cancer tissue. [file 13059_2025_3870_MOESM1_ESM.pdf]

Supplementary materials

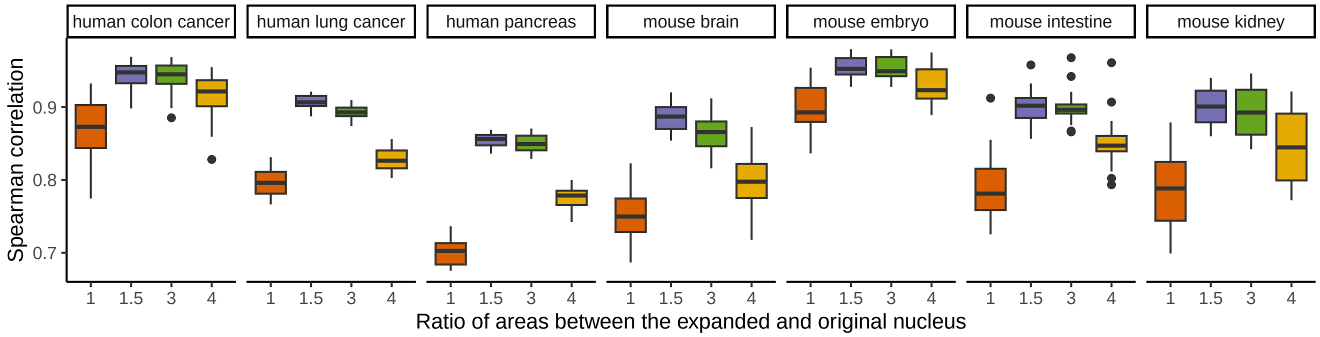

Figure S1. Spearman correlation coefficient between the overall gene rankings obtained by the default `ctSVG` and those obtained by `ctSVG` with different levels of cell nuclei expansion (x-axis).

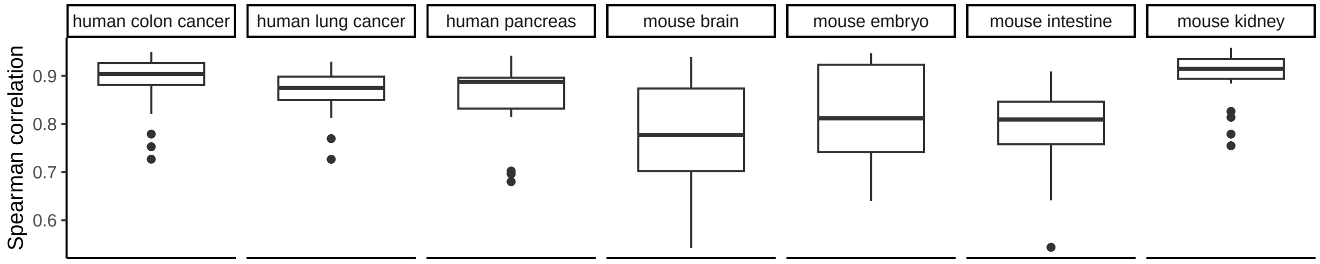

Figure S2. Spearman correlation coefficient between the overall gene rankings obtained by `ctSVG` from the original data and those from the perturbed data.

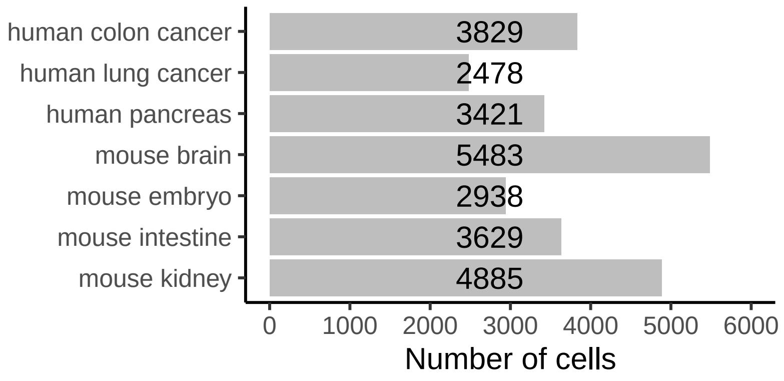

Figure S3. Number of cells in each Visium HD dataset after rectangular subsetting.

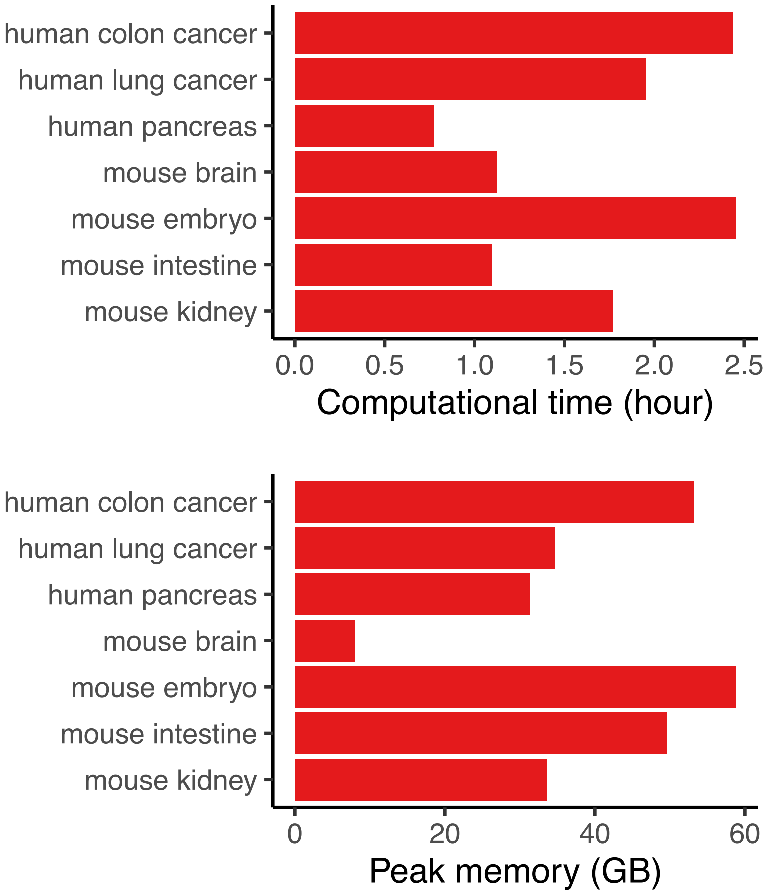

Figure S4. Computational time (top) and peak memory usage (bottom) of `ctSVG` across seven real Visium HD datasets.

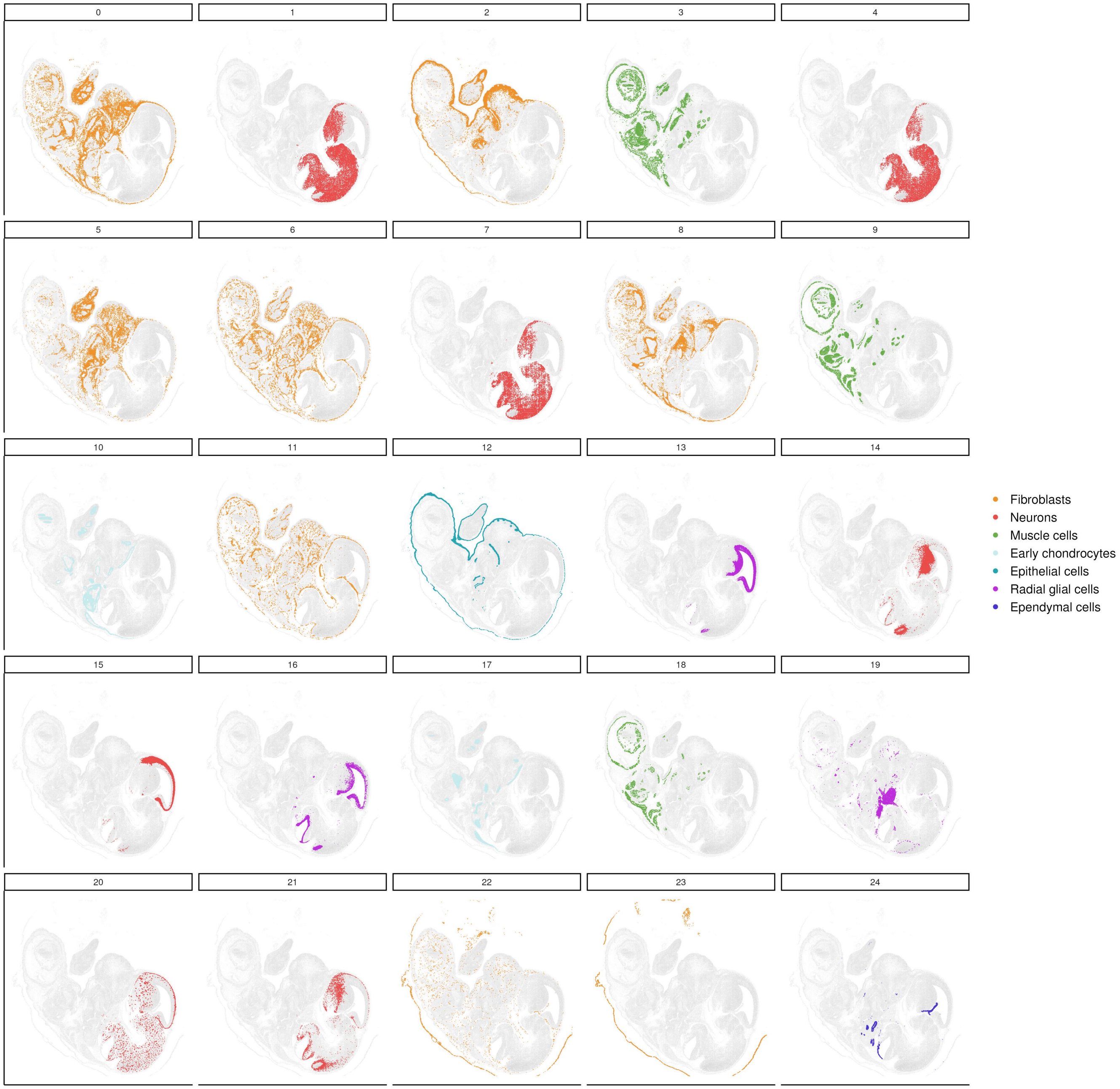

Figure S5. Cell clustering and cell type annotation in the mouse embryo tissue.

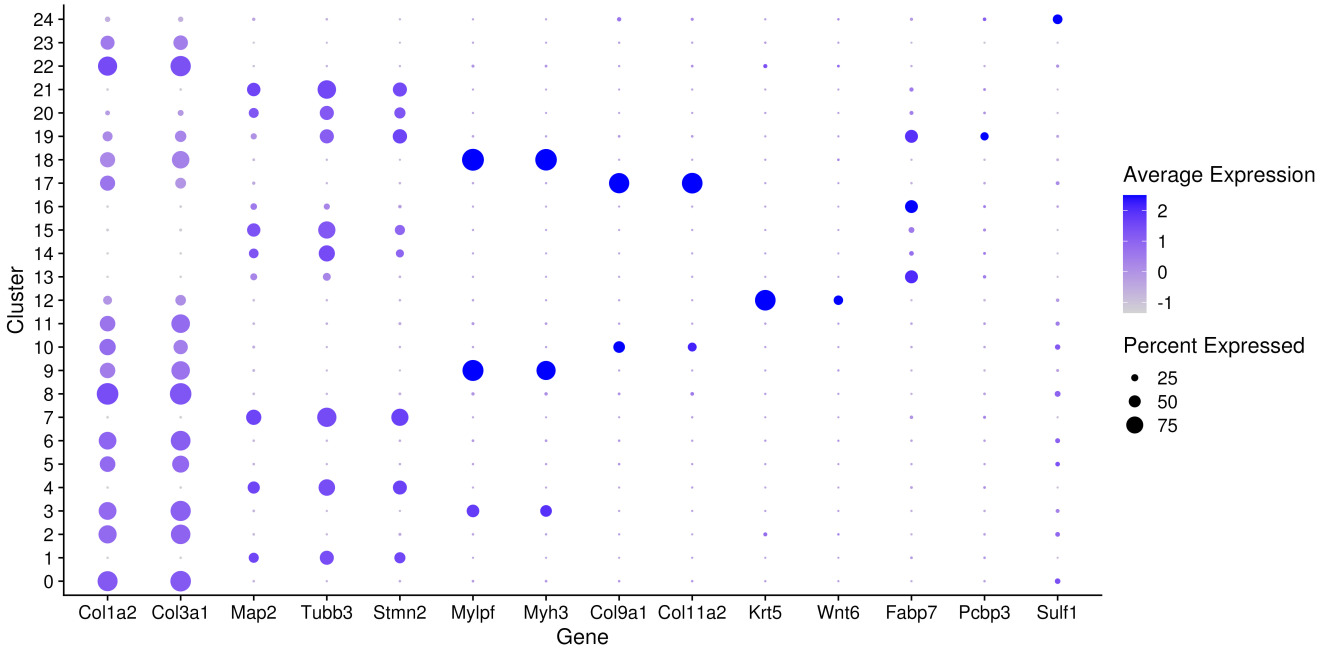

Figure S6. Expression level of marker genes in the mouse embryo tissue.

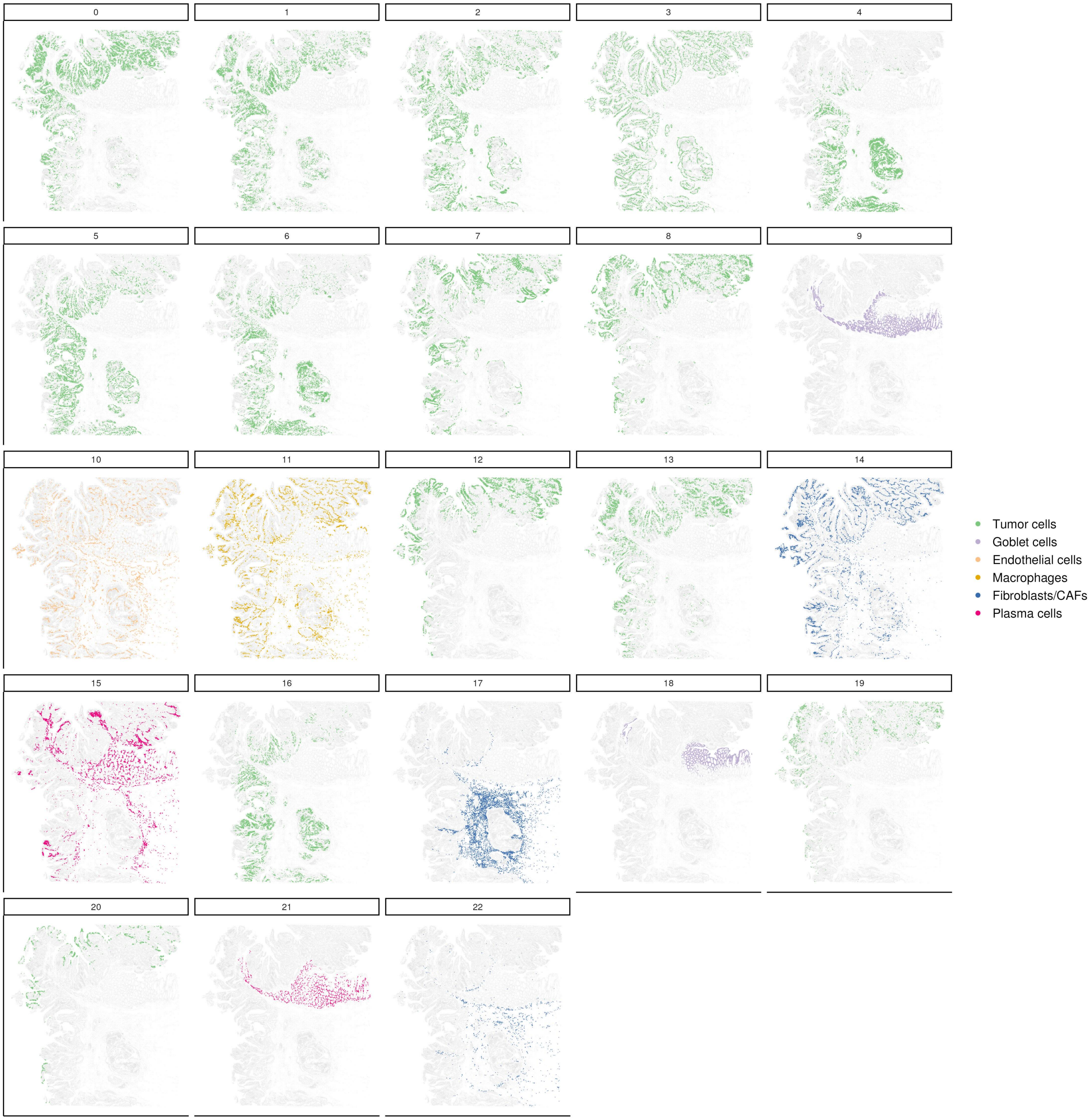

Figure S7. Cell clustering and cell type annotation in the human colon cancer tissue.

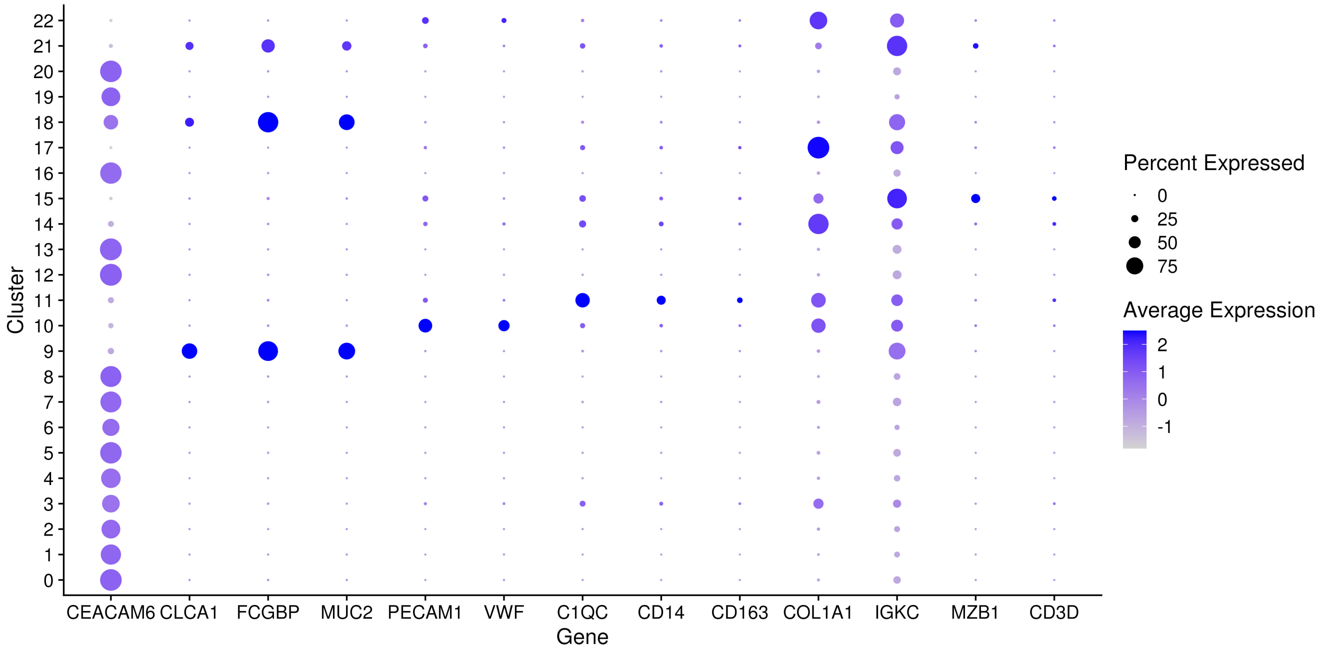

Figure S8. Expression level of marker genes in the human colon cancer tissue.
